# The Appearance and Modulation of Osteocyte Marker Expression during Calcification of Vascular Smooth Muscle Cells

**DOI:** 10.1371/journal.pone.0019595

**Published:** 2011-05-17

**Authors:** Dongxing Zhu, Neil Charles Wallace Mackenzie, José Luis Millán, Colin Farquharson, Vicky Elizabeth MacRae

**Affiliations:** 1 The Roslin Institute, The University of Edinburgh, Roslin, Midlothian, Scotland, United Kingdom; 2 Sanford Children's Health Research Center, Sanford-Burnham Medical Research Institute, La Jolla, California, United States of America; L' Istituto di Biomedicina ed Immunologia Molecolare, Consiglio Nazionale delle Ricerche, Italy

## Abstract

**Background:**

Vascular calcification is an indicator of elevated cardiovascular risk. Vascular smooth muscle cells (VSMCs), the predominant cell type involved in medial vascular calcification, can undergo phenotypic transition to both osteoblastic and chondrocytic cells within a calcifying environment.

**Methodology/Principal Findings:**

In the present study, using *in vitro* VSMC calcification studies in conjunction with *ex vivo* analyses of a mouse model of medial calcification, we show that vascular calcification is also associated with the expression of osteocyte phenotype markers. As controls, the terminal differentiation of murine calvarial osteoblasts into osteocytes was induced *in vitro* in the presence of calcifying medium (containing ß-glycerophosphate and ascorbic acid), as determined by increased expression of the osteocyte markers DMP-1, E11 and sclerostin. Culture of murine aortic VSMCs under identical conditions confirmed that the calcification of these cells can also be induced in similar calcifying medium. Calcified VSMCs had increased alkaline phosphatase activity and PiT-1 expression, which are recognized markers of vascular calcification. Expression of DMP-1, E11 and sclerostin was up-regulated during VSMC calcification *in vitro*. Increased protein expression of E11, an early osteocyte marker, and sclerostin, expressed by more mature osteocytes was also observed in the calcified media of *Enpp1^−/−^* mouse aortic tissue.

**Conclusions/Significance:**

This study has demonstrated the up-regulation of key osteocytic molecules during the vascular calcification process. A fuller understanding of the functional role of osteocyte formation and specifically sclerostin and E11 expression in the vascular calcification process may identify novel potential therapeutic strategies for clinical intervention.

## Introduction

Vascular calcification is a marker of increased cardiovascular risk in a number of diseases, including diabetes, atherosclerosis and end-stage renal disease [1]–[3]. The process of vascular calcification shares many similarities with that of bone formation [1], [2]. Chondrocytes and osteoblasts calcify their extracellular matrix (ECM) during endochondral bone formation by promoting the formation of crystalline hydroxyapatite, through a series of physico-chemical and biochemical processes.

Osteocytes are terminally differentiated osteoblasts and make up 90% of the cells present within bone. They are distinctive and isolated cells that are embedded within the bone matrix. Although the precise actions of osteocytes in bone have yet to be fully elucidated, these cells play pivotal mechanomodulatory roles in directing bone formation and bone resorption in response to load-bearing [4]. Osteocytes also have a reported role in mineral homeostasis. They are capable of modifying the matrix environment around them [5] and produce calcification modifying hormones and growth factors [6], [7]. There is also a requirement for the local production of matrix metalloproteinases and modulators of calcification in the osteocyte's canalicular–lacuna environment for healthy osteocyte function [8].

Within the past two decades, a number of osteocyte markers have been identified, including dentin matrix protein-1 (DMP-1), E11 and sclerostin [6], [7]. DMP-1 is an ECM protein member of the SIBLING family and recent work has emphasized the relative osteocyte specificity of DMP-1 and implicated it in osteocyte function and signalling [9]. DMP-1 is known to be produced in response to mechanical loading [10] and DMP-1 ablation induces a hypomineralized phenotype associated with elevated levels of circulating FGF-23 and defective osteocyte network formation [11]. DMP-1 produced by the osteocyte regulates FGF-23 production, and acts through a bone-kidney axis to control phosphate homeostasis [11].

E11, also called podoplanin, OTS-8, gp38, or PA2.25 based on its identification in different tissues, is a mucin-type glycoprotein with O-glycosylation and high sialic acid content [12]. E11 is highly expressed in osteocytes that are in the process of embedding within the ECM or have recently embedded [13]. It is also expressed in several other cell types with a dendritic morphology, including kidney podocytes, type II lung alveolar cells, and cells of the choroid plexus [7]. Indeed, a functional link between E11 expression and osteocyte dendrite formation was evident in osteoblasts in which E11 expression had been reduced. This gene silencing approach resulted in the inhibitition of cytoplasmic processes development [13]. E11 has also been proposed to function in the adhesion of cells to the bone matrix [14].

More mature deeply embedded osteocytes express high levels of sclerostin, the glycoprotein product of the *SOST* gene [15]. Loss-of-function mutations in *SOST* cause progressive bone overgrowth in humans [16]. *SOST*-null mice also have a high bone mass phenotype [17]. These negative effects of sclerostin on osteoblastogenesis and bone formation are most likely exerted through its antagonizing effects on LRP5 actions, a key activator of the Wnt/β-catenin signalling pathway [15], [18].

A number of studies have reported that vascular smooth muscle cells (VSMCs), the predominant cell type involved in vascular calcification, can undergo phenotypic transition to osteoblastic and chondrocytic cells in a calcified environment [19]–[23]. Cells of an osteocyte phenotype have been observed in Mönckeberg's sclerosis lesions, which are characterized by medial calcification of peripheral arteries [24]. However, it has yet to be established whether VSMC calcification involves a transition to an osteocyte phenotype. Therefore, in the present study we have undertaken *in vitro* VSMC calcification studies, in conjunction with *ex vivo* analyses of a mouse model of medial calcification and demonstrated that vascular calcification is associated with the appearance of an osteocyte phenotype.

## Materials and Methods

### Ethics statement

All animal experiments were approved by The Roslin Institute's Animal Users Committee and the animals were maintained in accordance with Home Office guidelines for the care and use of laboratory animals.

### Primary murine calvarial osteoblast isolation

Primary mouse osteoblasts were obtained by sequential enzyme digestion of excised calvarial bones from 3-d-old wild-type C57BL/6 mice using a four-step process [1 mg/ml collagenase type II in Hanks' balanced salt solution (HBSS) for 10 min; 1 mg/ml collagenase type II in HBSS for 30 min; 4 mM ethylenediaminetetraacetic acid (EDTA) for 10 min; 1 mg/ml collagenase type II in HBSS for 30 min]. The first digest was discarded and the cells subsequently obtained were resuspended in growth medium consisting of α-MEM (Invitrogen, Paisley, UK) supplemented with 10% FCS (Invitrogen) and 1% gentamycin (Invitrogen). Cells were cultured for 4 d in a humidified atmosphere of 95% air/5% CO_2_ at 37°C in T75 tissue culture flasks (Greiner Bio-One, GmbH, Frickenhausen, Baden-Württemberg, Germany) until confluent.

### Primary murine VSMC isolation

Primary VSMCs were isolated from aortae dissected from wild-type C57BL/6 mice at 5 wks of age. The adventitia was removed and the aorta cut open to expose the endothelial layer [22]. Tissues from eight animals were pooled for digestion with 1 mg/ml trypsin for 10 min to remove any remaining adventitia and endothelium. This was followed by incubation overnight at 37°C in a humidified atmosphere of 95% air/5% CO_2_ in growth medium. Tissues were then digested in 425 U/ml collagenase type II for 5 h. Before experimentation, isolated VSMCs were expanded in growth medium for two passages in T25 tissue culture flasks (Greiner Bio-One) coated with 0.25 µg/cm^2^ murine laminin (Sigma, Poole, UK) to promote maintenance of the contractile differentiation state [25].

### Primary murine osteoblast and VSMC culture

Osteoblasts and VSMCs were seeded at a density of 1.5×10^4^ cells/cm^2^. At confluency (day 0), growth medium was supplemented with 2.5 mM ß-glycerophosphate (ßGP) and 50 µg/ml ascorbic acid for up to 28 d to induce matrix calcification. Incubation was at 37°C in a humidified atmosphere of 95% air/5% CO_2_ and the medium was changed every second/third day.

### Detection of calcification

Calcium deposition was evaluated by staining the cell-matrix monolayer with alizarin red [26], [27]. Cells were washed twice with phosphate buffered saline (PBS), fixed in 4% paraformaldehyde for 5 min at 4°C, stained with 2% alizarin red (pH 4.2) for 5 min at room temperature and rinsed with distilled water. Alizarin red stained cultures were extracted with 10% cetylpyridium chloride for 10 min and the O.D. was determined at 570 nm by spectrophotometery (Multiskan Ascent, Thermo Electron Corporation, Vantaa, Finland).

The matrix was also decalcified in 0.6 N HCL for 24 h, and free calcium determined colorimetrically using a commercially available kit (Randox Laboratories Ltd, Crumlin, County Antrim, UK) and corrected for total protein concentration. The protein content of the cultures was measured using the Bio-Rad protein assay reagent (Bio-Rad Laboratories, Hertfordshire, UK) based on the Bradford dye binding procedure, and gamma globulin was used as standard [28].

### Alkaline Phosphatase (ALP) activity

Cell layers were lysed with 0·9% NaCl and 0·2% Triton X-100 and centrifuged at 12 000 g for 15 min at 4°C. The supernatant was assayed for protein content and ALP activity. Enzyme activity was determined by measuring the cleavage of 10 mM p-nitrophenyl phosphate (pNPP) at 410 nm using a commercially available kit (Thermo Trace, Melbourne, Australia). Total ALP activity was expressed as nmoles pNPP hydrolysed/min/mg protein [29].

### Analysis of gene expression using quantitative RT-PCR

RNA was extracted from cells using RNeasy total RNA (Qiagen Ltd, Crawley, West Sussex, UK), according to the manufacturer's instructions. For each sample, total RNA content was assessed by absorbance at 260 nm and purity by A260/A280 ratios. RNA was reverse transcribed and the PCR reaction undertaken as described previously [30], [31]. All genes were analyzed with the SYBR green detection method using the Stratagene Mx3000P real-time QPCR system (Stratagene, CA, USA). Each PCR was run in triplicate. All gene expression data were normalized against *Gapdh* and the control values expressed as 1 to indicate a precise fold change value for each gene of interest. Primers for *PiT-1* (Forward 5′CAC TCA TGT CCA TCT CAG ACT3′, Reverse 5′CGT GCC AAA GAA GGT GAA C3′), *ALP* (*Akp2*) (Forward 5′GGG ACG AAT CTC AGG GTA CA3′, Reverse 5′AGT AAC TGG GGT CTC TCT CTT T3′), *E11* (Forward 5′AAC AAG TCA CCC CAA TAG AGA TAA T3′ Reverse 5′CTA ACA AGA CGC CAA CTA TGA TTC3′), *SOST*, *Dmp-1* (Qiagen; sequence not disclosed) and *Gapdh* (Primer Design, Southampton, UK; sequence not disclosed) were used.

### Western blotting

Cultured cells were lysed in RIPA buffer (Invitrogen) containing “complete” protease inhibitor cocktail according to manufacture's instructions (Roche, East Sussex, UK). Immunoblotting was undertaken as previously described [27], [31]. Nitrocellulose membranes were probed overnight at 4°C with anti-sclerostin or anti-E11 primary antibody (1∶1000 dilution in 5% BSA), (R&D Systems, Abingdon, UK), washed in TBST and incubated with anti-goat IgG-peroxidase (DAKO, Glostrup, Denmark) for 1 h (1∶1000 dilution in 5% milk). The immune complexes were visualised by enhanced chemiluminescence (ECL) (GE Healthcare, Buckinghamshire, UK). Membranes were then washed in ‘stripping buffer’ (Pierce, Rockford, Il, USA) and re-probed for 1 h for ß-actin expression (1∶5000 dilution in 5% milk; anti ß-actin clone AC15; Sigma). After washing, membranes were incubated with anti-mouse IgG-peroxidase for 1 h (Sigma).

### Maintenance of *Enpp1^−/−^* mice

The generation and characterization of the ectonucleotide pyrophosphatase/phosphodiesterase 1 null (*Enpp1* ^−/−^) mouse has been previously described [32]. To determine genotypes, genomic DNA was isolated from ear clips and analyzed using PCR protocols developed by Genetyper (Genetyper, New York, USA).

### Immunohistochemistry

Tibiae and aortae were dissected from 22 week-old *Enpp1* ^−/−^ and *Enpp1* ^+/+^ wild-type mice that had been euthanized. After fixation in 70% ethanol the tibiae were decalcified in 10% EDTA (pH 8.0) for 4 wks at 4°C. Tissues were finally dehydrated and embedded in paraffin wax before sectioning at 5 µm (tibiae) or 4 µm (aortae) using standard procedures. For histological analysis, sections were de-waxed in xylene and antigen retrieval was achieved by treatment with 0.1% trypsin for 10 min. Endogenous peroxidises and non-specific antibody binding were blocked before overnight incubation at 4°C with 0.5 µg IgG/ml anti-sclerostin or anti-E11 antibodies (R&D Systems). The slides were then washed in PBS, and incubated with rabbit anti-goat IgG peroxidise (1∶200 dilution) using the Vectastain ABC kit (Vector Laboratories, Peterborough, UK) following the manufacturer's instructions. The sections were finally dehydrated, counterstained with haematoxylin and eosin and mounted in DePeX. Control sections were incubated with non-immune goat IgG (0.5 µg IgG/ml) in place of the primary antibody.

### Statistical analysis

General Linear Model analysis and the Students t-test were used to assess the data. All data are expressed as the mean+/−S.E.M. Statistical analysis was performed using Minitab 15. *P*<0.05 was considered to be significant.

## Results

### Differentiation of osteoblasts into osteocytes *in vitro*

**Figure 1:**
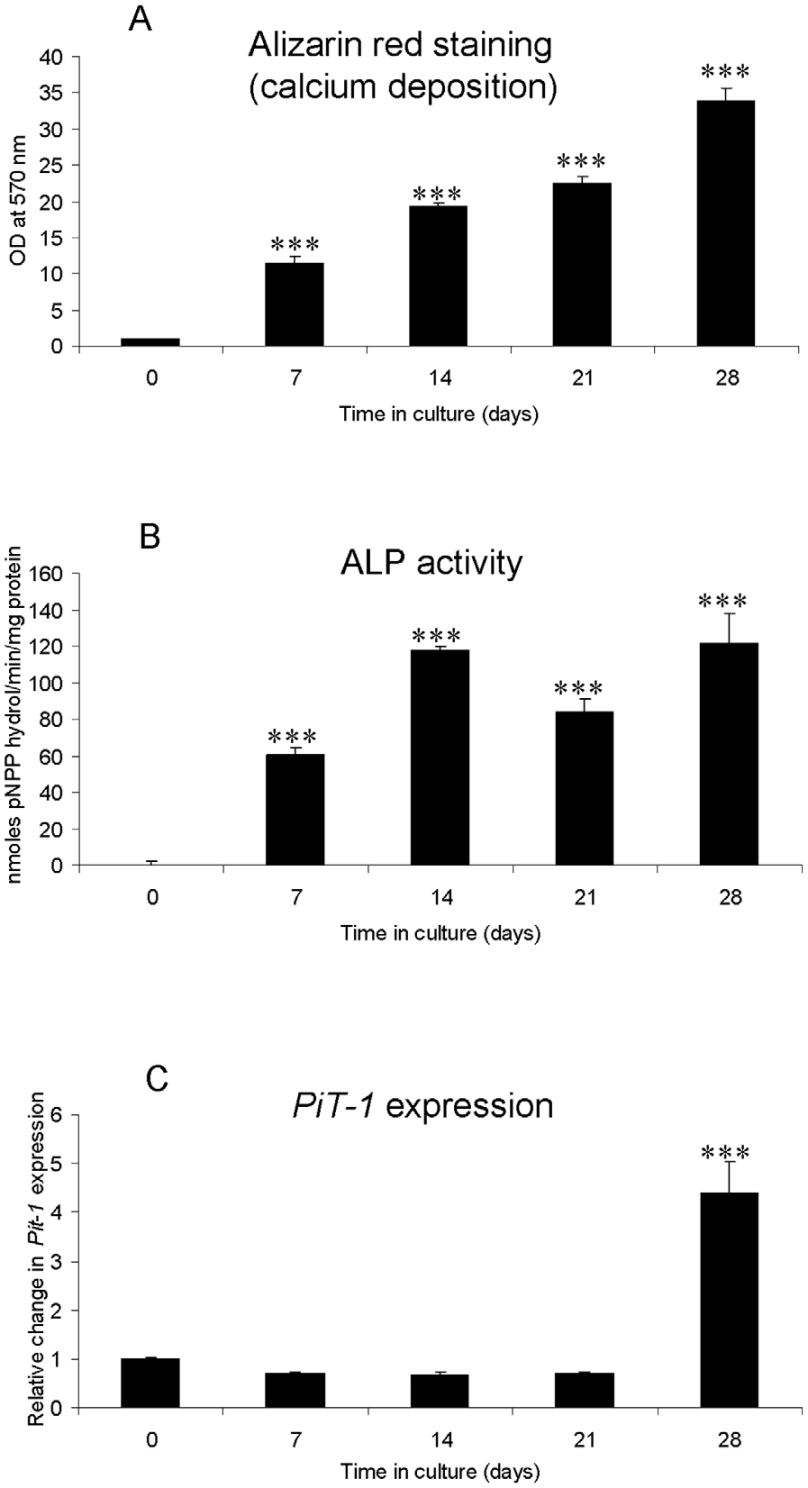
*In vitro* calcification of murine calvarial osteoblast cells cultured for 28 d under calcifying conditions. Quantification of (a) alizarin red staining (calcium deposition) and (b) Alkaline phosphatase activity (mean moles pNPP hydrol/min/mg protein). (c) Fold changes in *PiT-1* mRNA expression in cultured osteoblasts. Results are presented as mean+/−S.E.M. *** P<0.001 compared with day 0.

Calvarial osteoblasts at confluency had negligible amounts of alizarin red staining (calcium deposition) (Fig. 1a) and ALP activity (Fig. 1b). Further culture in calcifying conditions for an additional 7, 14, 21 and 28 d resulted in significant increases in both ALP activity and alizarin red staining. In addition, mRNA expression of the type III sodium-dependent phosphate transporter *PiT-1*, a gene associated with osteoblast mineralization, was significantly increased at the end of the culture period (Fig. 1c). These data confirm the formation of calcified matrix in calvarial osteoblasts over the 28-day culture period.

**Figure 2:**
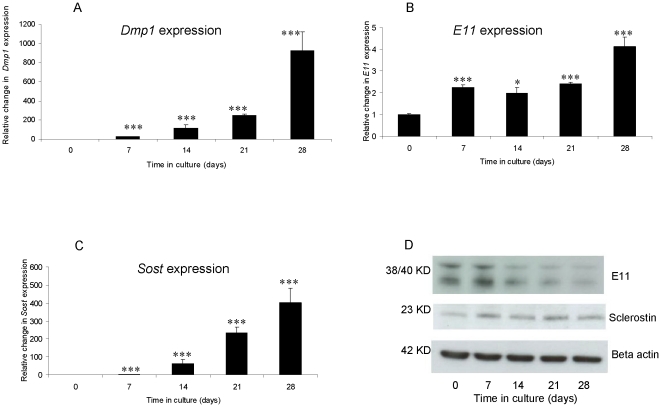
Up-regulation of osteocyte markers during *in vitro* calcification of murine calvarial osteoblast cells cultured for 28 d under calcifying conditions. Fold changes in mRNA expression of (a) *Dmp-1*, (b) *SOST* and (c) *E11*. (d) Sclerostin and E11 protein expression in corresponding cultured osteoblasts. Results are presented as mean+/−S.E.M. * P<0.05; *** P<0.001 compared with day 0.

mRNA expression of *Dmp-1* and *E11* was observed at all time points during the culture period. By 7 d, a significant increase in both *Dmp-1* (29.5 fold; P<0.001); Fig. 2a) and *E11* (2.2 fold; P<0.001); Fig. 2b) mRNA expression was noted. This increased expression was maintained throughout the culture period. *SOST* expression was also observed at all time points, with a significant increase in expression also seen by 7 d (3.4 fold; P<0.001; Fig. 2c). Comparable changes in sclerostin protein expression were observed, however E11 protein expression appeared to reduce following 21d in culture (Fig. 2d), which may be associated with post-transcriptional or post-translational regulation of expression. Previous studies have reported that E11 protein is degraded by the calpain family of proteinases [33]. These expression studies demonstrate the terminal differentiation of calvarial osteoblasts into the osteocyte phenotype *in vitro*.

### VSMC calcification *in vitro* is associated with an osteocyte phenotype

**Figure 3:**
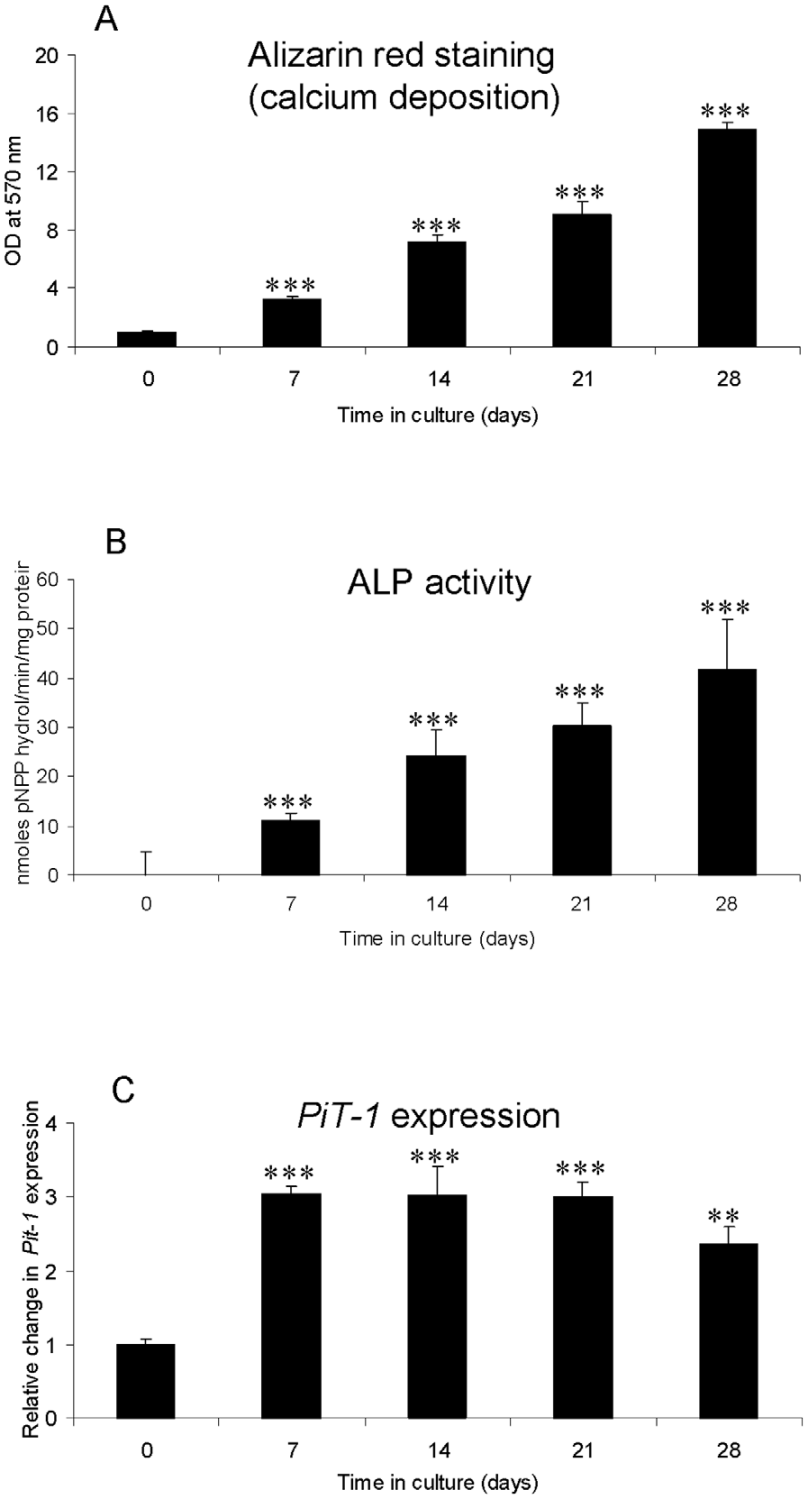
*In vitro* calcification of murine aortic VSMCs cultured for 28 d under calcifying conditions. Quantification of (a) alizarin red staining (calcium deposition) and (b) Alkaline phosphatase activity (mean moles pNPP hydrol/min/mg protein). (c) Fold changes in *PiT-1* mRNA expression in cultured VSMCs. Results are presented as mean+/−S.E.M. ** P<0.01; ***P<0.001 compared with day 0.

Alizarin red staining (calcium deposition) (Fig. 3a) and ALP activity (Fig. 3b) in murine aortic VSMCs were negligible at 0 d of culture. Significant increases in both matrix mineralization and ALP activity were noted following 7, 14, 21 and 28 d of culture in calcifying medium. A significant increase in mRNA expression of *PiT-1* was seen by 7 d (3.0 fold; P<0.001; Fig. 3c). These results confirm the *in vitro* calcification of aortic VSMCs over the 28-day culture period.

**Figure 4:**
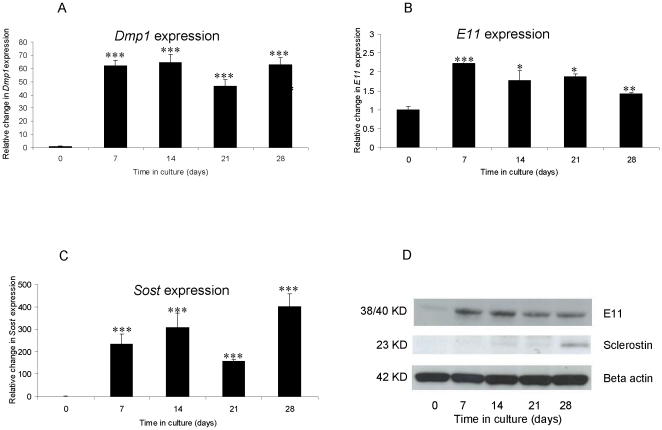
Up-regulation of osteocyte markers during *in vitro* calcification of murine aortic VSMCs cultured for 28 d under calcifying conditions. Fold changes in mRNA expression of (a) *Dmp-1*, (b) *SOST* and (c) *E11*. (d) Sclerostin and E11 protein expression in corresponding cultured osteoblasts. Results are presented as mean+/−S.E.M. * P<0.05; ** P<0.01; *** P<0.001 compared with day 0.

*Dmp-1* and *E11* mRNA expression was noted at all time points. By 7 d, a significant increase in both *Dmp-1* (61.8 fold; P<0.001); Fig. 4a) and *E11* (2.2 fold; P<0.001); Fig. 4b) mRNA expression was observed, which was maintained throughout the 28 d culture period. *SOST* expression was also observed at all time points, with a significant increase in expression also seen by 7 d (234.8 fold; P<0.001; Fig. 4c). The temporal E11 protein expression mirrored that of *E11* gene expression with higher levels noted from day 7 onwards (Fig. 4d). Significant sclerostin protein expression was however only noted at day 28 of culture. (Fig. 4d). These studies show that calcification caused by elevated phosphate levels (through the addition of βGP) induces VSMCs to undergo an osteocytic phenotype transition *in vitro*.

**Figure 5:**
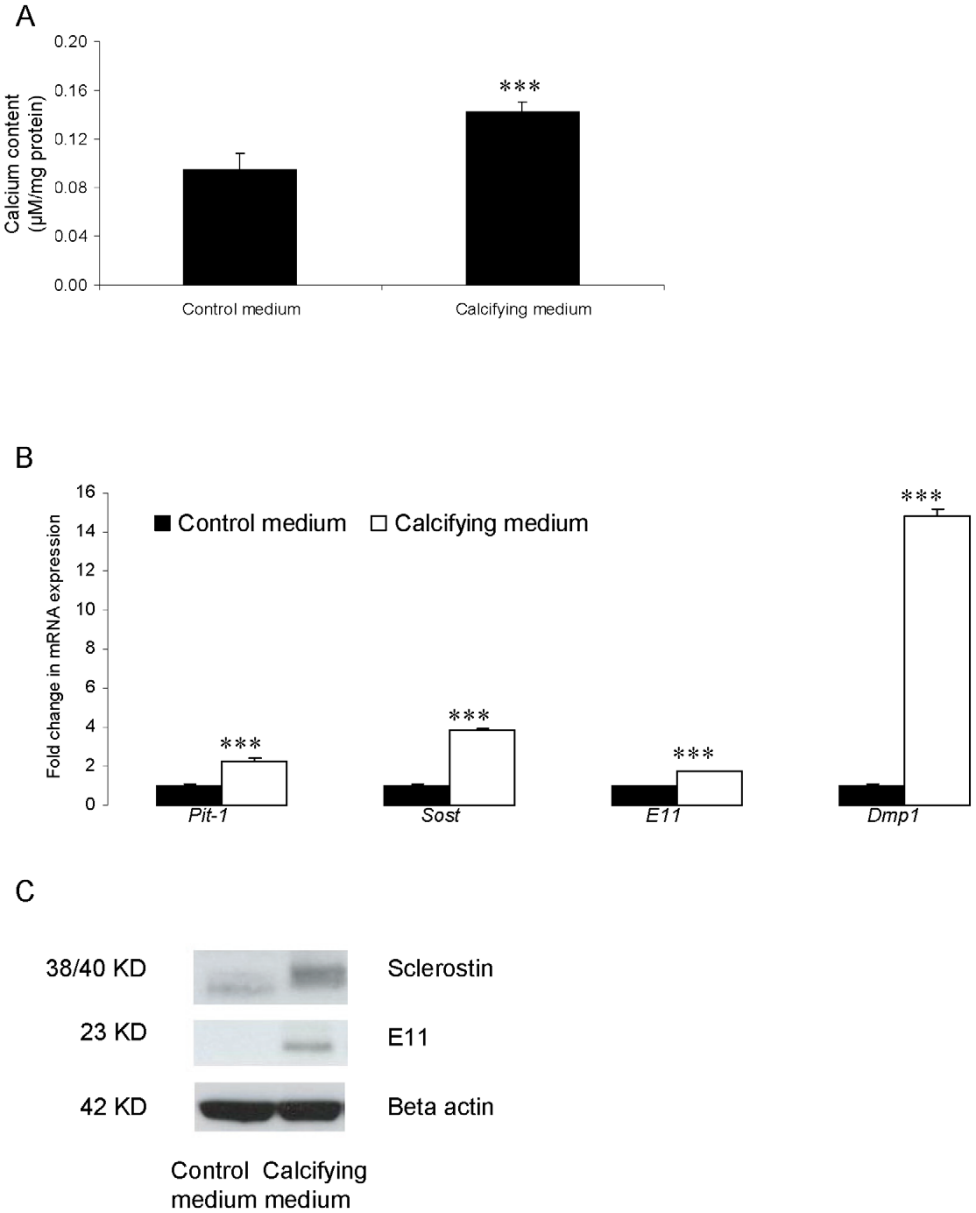
*In vitro* calcification of murine aortic VSMCs cultured for 21 d in the presence or absence of ßGP. (a) Calcium content (µM/mg protein). (b) Fold changes in mRNA expression of *PiT-1*, *SOST*, *E11* and *Dmp-1*. (c) Sclerostin and E11 protein expression in corresponding cultured VSMCs. Results are presented as mean+/−S.E.M. * P<0.05; *** P<0.001 compared with absence of ßGP.

Further examination of aortic VSMC osteocyte marker expression during matrix calcification was achieved in a comparison of VSMCs maintained under calcifying and non-calcifying conditions. The calcium content (P<0.05; Fig. 5a) and *PiT-1* gene expression (P<0.001; Fig. 5b) of VSMCs cultured for 21 d in the presence of ßGP was significantly increased in comparison to non-calcifying control cells. Also, significant increases in mRNA (P<0.001; Fig.5b) and protein (Fig. 5c), expression of E11 and sclerostin expression were observed in the calcifying cultures. These studies further confirm that phosphate induced *in vitro* calcification of aortic VSMCS is associated with the expression of markers of the osteocyte phenotype.

### Osteocyte markers are present in the calcified aorta from the *Enpp1^−/−^* mouse

**Figure 6:**
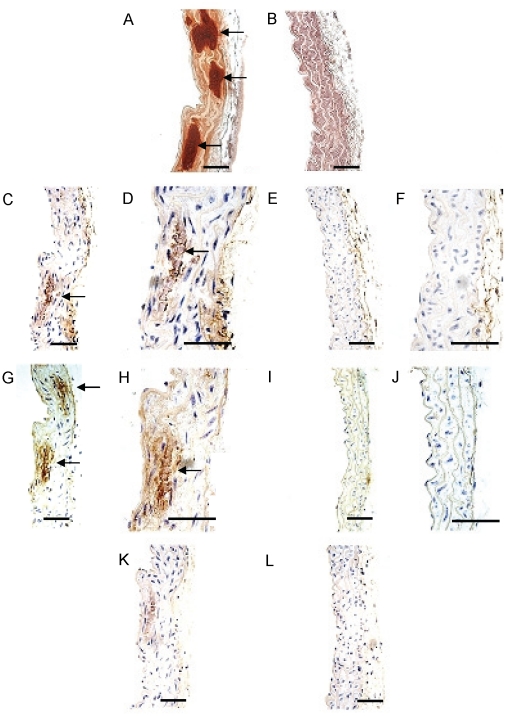
Emergence of osteocytic markers in the calcified aorta from the *Enpp1^−/−^* mouse *in vivo*. Medial aortic calcification was detected by alizarin red staining (arrows) in (a) *Enpp1^−/−^* tissue compared to (b) *Enpp1^+/+^* control. Increased protein expression of (c, d) E11 and (g, h) Sclerostin was observed in the calcified media of *Enpp1^−/−^* aortic tissue (arrows). No expression of (e, f) E11 and (i, j) Sclerostin was detected in the non-calcified media of the *Enpp1^+/+^* control. Representative images of (K) *Enpp1^−/−^* tissue and (l) *Enpp1^+/+^* negative control tissues. Scale bars = 50 µm.

**Figure 7:**
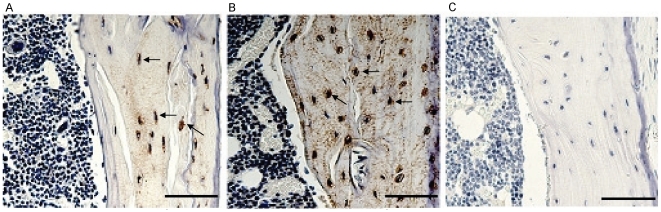
Confirmation of osteocytic markers in the mouse tibia *in vivo*. Protein expression of (a) Sclerostin and (b) E11 was confirmed in osteocytes of a positive control mouse tibia (arrows). (c) Representative image of appropriate negative control. Scale bars = 100 µm.

The association of an osteocyte phenotype with vascular calcification was further strengthened by examination of the *Enpp1^−/−^* mouse. This mouse model shows decreased levels of the mineralization inhibitor pyrophosphate, with phenotypic features including significant alterations in bone mineralization in long bones and calvariae, and pathologic, severe perispinal soft tissue and medial arterial calcification [32]. Calcification in the medial layer of the *Enpp1^−/−^* aorta was confirmed by alizarin red staining (Fig. 6a), with no staining observed in wild-type controls (Fig. 6b). Following confirmation of sclerostin (Fig. 7a) and E11 (Fig. 7b) protein expression in osteocytes and associated canaliculi within cortical bone, expression of both proteins was also detected in the *Enpp1^−/−^* calcified aortic media (Fig. 6c&d and Fig. 6g&h respectively). No positive staining for E11 or sclerostin was seen in wild-type mice (Fig. 6e&f and Fig. 6i&j respectively) or control sections incubated with IgG only (Fig. 6l). These immunolocalisation studies verify our *in vitro* data, and confirm the up-regulation of markers associated with an osteocyte phenotype during the vascular calcification process.

## Discussion

Despite the clinical importance of vascular calcification, the precise biochemical processes regulating this pathological process are not yet fully understood. A number of studies have shown that VSMCs, the predominant cell type involved in vascular calcification, can undergo phenotypic transition to osteoblastic and chondrocytic cells in a calcified environment [19]–[23]. However, it has yet to be established whether VSMC calcification is associated with the terminal differentiation of the nascent osteoblasts to an osteocyte phenotype. Such terminal differentiation would strongly imply that similar osteoblast phenotypic transitions observed in bone also occur in vascular tissue, which would be suggestive of common regulatory and differentiation pathways in both tissues.

In the present study, quantitative alizarin red staining of calcium deposition confirmed the formation of calcified matrix in murine calvarial osteoblast cultures, induced by treatment with ascorbic acid and ßGP. This is in agreement with previous reports [34], [35]. In addition, calvarial cells cultured under calcifying conditions showed increased ALP activity and expression of PiT-1 which are recognised regulators of osteoblast matrix calcification [34]–[36].

Culture of murine VSMCs under identical conditions confirmed that the calcification of the matrix of these cells can also be induced by ßGP and ascorbic acid. Calcified VSMC also showed increased ALP activity and PiT-1 expression, which are recognized regulators of vascular calcification. Interestingly, the temporal expression pattern of PiT-1 differed notably between the osteoblasts and VSMCs. This may be a result of PiT-1 being the predominant sodium-dependent phosphate co-transporter expressed in VSMCs [37]. Increased *PiT-1* expression leads to elevated intracellular phosphate and induces the osteogenic conversion of VSMCs [37]. Conversely, down-regulation of *PiT-1* expression by gene silencing has been shown to reduce phosphate uptake by VSMCs and inhibit phosphate-induced VSMC phenotypic transition and matrix calcification [37]. ALP has an important role in vascular calcification through its ability to generate phosphate, and by decreasing levels of the calcification inhibitor pyrophosphate in blood vessels and has been proposed as a therapeutic target for medial vascular calcification [34], [38].

In order to establish whether VSMC calcification involves a transition to an osteocyte phenotype, the expression patterns of DMP-1, E11 and sclerostin were investigated. Increased expression levels of *Dmp-1*, *E11* and *SOST* mRNA were noted after 7 days of culture in both osteoblasts and VSMCs. This increased expression was maintained throughout the culture period. However, temporal differences in sclerostin and E11 protein expression were observed between osteoblasts and VSMCs and this reflects the likelihood of osteoblasts more readily undergo this transistion compared to VSMCs. Interestingly E11 protein expression in osteoblasts appeared to reduce following 21 days in culture, which may be associated with post-transcriptional or post-translational regulation of expression.

These studies show that calcification caused by elevated phosphate levels induces VSMCs to undergo an osteocytic phenotype change. This is in agreement with studies in bone, which have shown that calcification drives osteocyte formation [39]. However, it has also been proposed that calcification may be directed by cells that are already differentiating into osteocyte-like cells [40]. Further imaging studies examining the calcification dynamics in VSMCs would establish whether calcification is directed by VSMCs undergoing an osteocytic phenotype change.

To our knowledge, this is the first report indicating that the osteocyte markers DMP-1, E11 and sclerostin are up-regulated during VSMC calcification *in vitro*. These *in vitro* data were confirmed and extended by studying an *in vivo* mouse model of vascular calcification. Mice lacking ecto-nucleotide pyrophosphatase/phosphodiesterases-1 (NPP1, a.k.a PC-1), a major generator of extracellular pyrophosphate, spontaneously develop articular cartilage, perispinal, and medial aortic calcification at a young age [41]. These NPP1 knockout mice (*Enpp1* ^−/−^) share phenotypic features with a human disease, idiopathic infantile arterial calcification [42], [43]. In the present study, our immunohistochemical approach demonstrated increased expression of both sclerostin and E11 in the calcified media of *Enpp1^−/−^* aortic tissue. This data is supported by the expression of sclerostin in CMV-Msx2 transgenic mice, which show extensive cardiovascular calcification [44]. Additionally, a comprehensive analysis of extracellular space components comprising the vascular proteome in humans recently identified the presence of sclerostin in aortic extracts [45]. Sclerostin has also been shown to bind to and influence the activity of the ECM protein, cysteine-rich protein 61 (Cyr61) [46]. Sclerostin potentiates Cyr61-mediated cell growth and vascular migration and alters Cyr61-mediated cellular adhesion [46]. Sclerostin may therefore play a key functional role in both physiological and pathological vascular tissue biology.

This study has demonstrated the up-regulation of molecules associated with the osteocyte phenotype in the *Enpp1* ^−/−^ mouse model of aortic medial calcification. Further studies are required to verify the phenotype transition of VSMCs to osteocytes in the vascular calcification process using additional pathological animal models, such as atherosclerosis and chronic kidney disease.

A fuller understanding of the importance of osteoblast terminal differentiation in this pathological process, and in particular functional studies to determine the role of sclerostin and E11, will lead to a better understanding of the etiology of VSMC calcification. Also, the identification of E11 as a potential driver of osteocytogenesis in vascular calcification may stimulate the development of novel potential therapeutic strategies for the inhibition of vascular calcification.
